# An ethnobotanical study of wild edible fruits in miombo woodlands of Tabora region in Western Tanzania

**DOI:** 10.1186/s13002-024-00668-x

**Published:** 2024-02-25

**Authors:** Michael Elias Mgalula

**Affiliations:** Mkwawa University College of Education, University of Dar es Salaam, Post Office Box 2513, Iringa, Tanzania

**Keywords:** Wild edible fruits, Ethnobotany, Indigenous knowledge, Utilisation, Cultural significance, Degradation threats

## Abstract

**Background:**

Wild edible fruits found in Tanzania's miombo woods are an indispensable source of food and medicine. Unfortunately, with the rapid expansion of human activities and urbanisation in the Tabora rural, Uyui and Sikonge districts of Western Tanzania, some wild fruits are disappearing due to the loss of plant diversity. The objectives of this study wereL: to document the knowledge related to wild edible fruits; to quantify the use and cultural significance, and to determine their threats.

**Methods:**

The ethnobotanical study was conducted from June 2022 to February 2023 involving 244 local informants. The study used field visits, the collection of plant parts, and semi-structured interviews with locals for its data collection. Descriptive statistics and correlation test were used to analyse the knowledge related to wild fruits. Frequency citation (*f*) and use reports (UR) were computed to understand the diversity and cultural significance (CI).

**Results:**

The study documented 27 wild edible fruit species used for food and medicine. The life form constituted deciduous shrubs or trees (64%), shrub trees (21%) and evergreen or deciduous trees (15%). About (56%) of wild edible fruits were collected from June to August after the rainy season, (33%) were harvested between December and May during the rainy season, and (11%) were gathered from September and November before the rainy season. Household size and sex of the respondents were significantly correlated to the knowledge of wild edible fruits. Higher utilisation frequency (*f*) was recorded for Vitex *mombassae* Vatke (*f* = 0.84), *Strychnos spinosa* Lam. (*f* = 0.82), *Vitex payos* (Lour.) Merr. (*f* = 0.56), *Phyllogeiton discolor* (Klotzsch) Herzog. (*f* = 0.45), *Vangueria infausta* Burchell (*f* = 0.45), *Tamarindus indica* L. (*f* = 0.38), *Parinari curatellifolia* (*f* = 0.25), *Landolphia parvifolia* K.Schum. (*f* = 0.22) and *Microcos conocarpa* Burret (*f* = 0.22) fruits species. Additionally, *Phyllogeiton discolor* (Klotzsch) Herzog. (UR = 56), *Vitex mombassae* Vatke (UR = 56), *Tamarindus indica* L. (UR = 37), *Strychnos spinosa* Lam. (UR = 14) and *Friesodielsia obovata* (Benth.) Verdc. (UR = 11), have higher use reports (UR) and considered culturally important. Wild fruits were used to cure diabetes, gastrointestinal, reproductive, and respiratory infections ailments.

**Discussion and conclusion:**

Multiple uses as well as the related knowledge of wild fruits have been documented. *Friesodielsia obovata*, *Grewia flavescens* Juss and *Thespesia garckeana* F.Hoffm. are the medicinal fruit species reported for the first time. Harvesting of wood plants, charcoal activities, crop cultivation, grazing expansion, and environmental change, have had an impact on the diversity of wild edible fruit plants. Over the past three decades, the use of wild fruits has been impacted by the loss of plant diversity due to decline of cultural norms on the forests management. Given the variety of uses for wild fruits, promoting markets for native fruits, sensitising the locals about the cultural importance and innovation on processing techniques are necessary to spur conservation efforts.

## Introduction

Wild edible fruits are essential food and medicine sources in many communities [1]. These fruit species are not cultivated but are grown spontaneously in their wild natural environments, such as forests and shrublands [2]. Wild fruits are essential to local diets and are a food source in all parts of the world that support the global food basket [3–5]. Unfortunately, wild edible fruits are characterised by limited development relative to their potential [6]. Traditional plant uses is declining in many communities in the world [7]. This declining is caused by alteration or disappearance of rural ecosystems in conserving forests or landscapes, many younger generations tend to ignore these native fruits [8], and increased reliance on exotic fruits and modern medicines [9]. Declining of cultural practice of wild fruits would inevitably lead to loss of indigenous knowledge of plant use, potentially affecting traditional plant knowledge into future [7]. In many rural communities worldwide, the use of wild fruits remains important because rural families do not have access to modern or exotic fruits. If they are available, they are relatively expensive [10]. As a result, most rural families in many developing countries remain undernourished because they do not have a variety of food to meet their daily requirements, and many more people are deficient in one or more micronutrients [11]. It is estimated that nearly 800 million people are chronically hungry, and 2 billion individuals are undernourished due to micronutrient deficiencies [12]. Consuming wild edible fruits can be a good source of vitamins and supplement micronutrient deficiencies [13, 14]. The diversity of wild edible fruits across different ecological zones in Africa, such as miombo woodlands is essential because wild edible fruits are rich sources of vitamins, minerals and valuable phytochemicals [15].

Wild fruits are the most widely known non-timber forest products (NTFPs) [16]. If well utilised, they can supplement diet, medicine, and material substance and are important sources of income essential for purchasing required household goods in rural settings [17, 18]. However, ever-increasing demands for land resources to produce food and the privatisation of land altogether have resulted in the degradation of natural ecosystems due to deforestation [19]. While those dynamics are happening, there is much concern about increasing global hunger due to a rising human population and urbanisation on the one hand and, on the other, climate variability, which is exponentially increasing. These stresses suppress the availability and utilisation of wild edible fruits in most rural communities in the sub-Saharan Africa. As a result, the use of wild fruits as food has declined mainly except for a few rural communities who continue utilising these indigenous fruits in their natural habitats [8].

In Eastern Africa, studies have documented numerous indigenous plants; coupled with [20, 21] nearly 3–8% of the estimated 7000 higher plants of Ethiopia are edible, 25% of which are cultivated. There are also many wild edible plants (WEPs) that produce quantities of food. In Kenya, the country is endowed with nearly 6293 indigenous plants [22], including 800 are food [23]. Nevertheless, there are more unrecorded wild plant species that thought to be edible [20]. Certainly, there is insufficient documentation about the diversity of wild fruits. Recording the knowledge about diversity and changes of untamed wild fruits can improve our knowledge related to food security and conservation in these countries [24].

In Tanzania, over 12,000 species of higher plants have been reported, and about 10% are estimated to be used as medicines to treat different human health conditions [25]. However, few ethnobotanical studies have been conducted to analyse the diversity of wild edible fruits, their uses, cultural significance and the threats, especially from the miombo woodlands of Tanzania. Miombo woodlands of Tanzania are known for their diversity of plant species [26]. However, most ethnobotanicals have slightly documented the cultural significance of wild edible fruits, including diversity and uses. Despite this lacuna, wild fruit species are continuing to become rare. Studies such as [27] reported on the nutritional composition and antioxidant properties of four species of wild edible fruits in southern Tanzania, and [28] reviewed usable wild plant species in relation to elevation and land use in mountainous areas of Kilimanjaro in Tanzania. Few studies have documented Tanzania's availability, preference, and consumption of wild edible fruits [29–33]. However, studies on the cultural significance of wild fruits and the threats due to human modifications are missing, especially in the miombo woodlands of western Tanzania. Given that a larger portion of Tanzania's western regions has extensive miombo woodlands with potential wild edible fruits, it is imperative to document the traditional knowledge about wild edible fruits in the context of societal and environmental change.

Traditionally, plants bearing edible fruits have been purposefully retained during land clearing for crop fields’ expansion or charcoal extraction [34], because they are food sources and can provide cash earnings. However, the growing disruptions caused by human activities continue to infringe on the variety of wild edible fruits. Research has indicated that while some wild edible plants adapt well to disturbances like grazing and plant harvesting [35, 36] or fire [37], others are more susceptible to changes in land use. Unlike modern fruit trees, which are more susceptible to climate change or land use change, wild edible trees possess adaptive traits that increase their resilience to stresses. To address this gap, the present study interviewed the local inhabitants of the districts of the Tabora region to document the knowledge about the diversity of wild edible fruits, uses and their cultural significance. The main aim of this ethnobotanical study is to document the diversity of wild edible fruits, their utilisation, cultural significance and threats facing wild edible fruits in the miombo woodlands of Tabora rural, Uyui and Sikonge districts in Tabora region of western Tanzania. The specific objectives are: (1) to document the knowledge about the diversity and seasonal availability of wild edible fruits; (2) to quantify utilisation frequency, use reports and cultural significance of wild fruits; (3) to determine the threats to wild edible fruits and conservation options.

## Materials and methods

### Description of the study areas

**Fig. 1:**
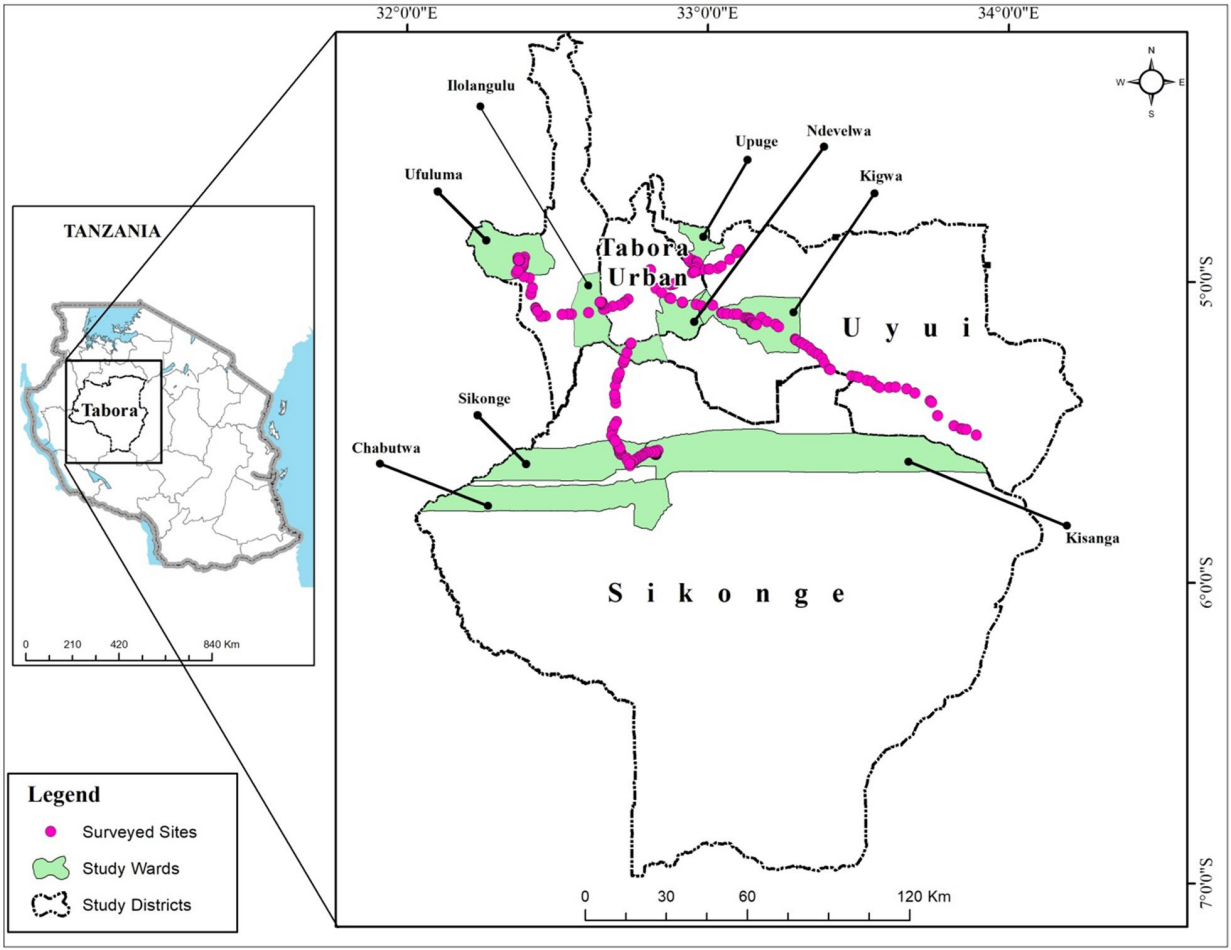
Location map of the three surveyed districts

The study was conducted in the Tabora region, located in Western Tanzania (Fig. 1). Three districts, namely Tabora Rural, Uyui, and Sikonge lie between latitude 3° 00′–7° 00′ South and longitude 32° 00′–34° 00′ East were surveyed. The region's elevation lies at 1000–1500 m above sea level, with lowlands and hilly areas rising to 1800 m above sea level [34]. The districts surveyed feature three agroecological zones based on their topography, which varies from low-lying plains, flat to undulating with isolated hills and ridges with outcrops of more resistant basement rocks. The climate is warm, and the mean annual temperature is around 23 °C, with a maximum monthly temperature varying between 27 and 30 °C, while the minimum monthly temperature varies from 15 to 18 °C [33]. The region's cooler months begin from May to July, with warmer periods from August to November. Rainfall is unimodal, occurring from November to May during the wet season while from June to October is the dry season. The studied locations exhibit slight variations in rainfall.

In the Western Uyui districts, North West Sikonge districts, and Tabora rural, the average annual rainfall is over 1000 mm, but decreases to 700 mm or less in the Eastern Uyui and Northern Sikonge districts [34]. Soils are 80–90% sand (ferric acrisol), with low organic carbon ranging between 0.4 and 0.8%. Vegetation community varied across the study locations; Sikonge district is endowed with miombo woodlands dominated by Brachystegia species, as well as Isoberlinia angolensis, Julbernardia globiflora, or Julbernardia paniculata. Some *Acacia and Combretum* species [38] are interspersed with other plants, bushland thicket interspersed with grassy grasslands, riverine and wetland vegetation. In Tabora rural, two conserved forest reserves are covered by miombo or interspersed with other vegetation communities, bushland thickets interspersed with grassy, wetland vegetation. In the Uyui district, a more significant portion of its land is dominated by bushland thicket, lowland grassy, wetland vegetation, miombo woodlands and wetland vegetation. The closed miombo woodland is protected as game and forest reserves.

The people in the study locations have diverse livelihood activities. The main socio-economic activities include crop farming, livestock keeping [33], and beekeeping [39]. Farmed crops include cash (tobacco, groundnut and sunflower) and subsistence (maize, sweet potatoes, cassava, millet, rice, and beans). Maize, sweet potatoes, cassava and rice are the main staple foods in the study locations. The extraction of firewood, charcoal production and petty business are other activities in the research areas. The ethnic groups in the study locations include the majority Nyamwezi, who speak Kinyamwezi; the Sukuma, who speak Kisukuma; and the minority Ha, who speak Kiha, but the official language spoken by the majority is Kiswahili. Most inhabitants in the studied locations are Christians or Muslims, with the numbers almost balanced, and very few people practice traditional worship. Since ancient time, wild plants have played an essential nutritional role in human survival in Tabora regions and medicinal applications.

### Ethnobotanical information

The study collected ethnobotanical data through semi-structured interviews and questionnaires from June 2022 through February 2023, which targeted key informants, herders, farmers, beekeepers and the people engaged in wood plant harvesting and charcoal extraction. A total of 244 respondents from eight (8) wards: Ndevelwa in Tabora Rural, Ufuluma, Ilolangulu, Upuge and Kigwa in Uyui district and Sikonge, Kisanga and Chabutwa in Sikonge district with ages ranging from 18 to 70 + were involved in the surveys (Table 1). During data collection, the researcher asked participants to describe habitats where wild edible fruits grow, their availability, uses and degradation threats. The questions about wild edible fruits mainly focused on local or vernacular names. The specimens of all 27 wild edible fruits recorded including parts of pants and the leaves were collected in the study sites by their vernacular names. The National Forestry Resources Monitoring and Assessment of Tanzania [40] book was referred to guide identification and documentation. Species identification was restricted to trees and shrubs, where participants identified trees and shrub species, including edible fruits grown in their environment. The botanist at the College of Natural and Applied Sciences (CONAS) of the University of Dar es Salaam (UDSM) in Tanzania identified all specimens and give the voucher numbers. The specimens of all 27 species of wild edible fruits are stored at the Department of Botany herbarium of the University of Dar es Salaam, Tanzania.

**Table 1:** Study villages, locations, elevation and number of households interviewed

Districts	Wards	Villages	GPS location		Elevation (m)	Total households	Households interviewed	Percent
Tabora rural	Ndevelwa	Inara	5° 05′ 53″ S	32° 52′ 29″ E	1216.27	221	30	12.3
Uyui	Kigwa	Kalofya- Ng'ambo	5° 08′ 59″ S	33° 07′ 54″ E	1238.96	350	30	12.3
	Upuge	Kasenga	4° 55′ 11″ S	32° 56′ 03″ ″E	1228.29	221	30	12.3
	Ilolangulu	Mpenge	3° 18′ 32″ S	32° 05′ 21″ E	1226.65	258	31	12.7
	Ufuluma	Umanda	4° 56′ 38″ S	32° 23′ 08″ E	1139.55	92	28	11.5
Sikonge	Sikonge	Mkolye- Isunda	5° 33′ 22″ S	32° 40′ 14″ E	1167.83	306	30	12.3
	Kisanga	Chag'ombe	5° 33′ 38″ S	32° 52′ 52″ E	1240.13	200	32	13.1
	Chabutwa	Kikungu	5° 58′ 59″ S	34° 06′ 59″ E	1487.01	320	33	13.5
							244	100

### Selection and sampling of the study areas

The study deployed purposive and stratified sampling techniques in order to guarantee representation from each important strata based on the presence of forest reserves such as Kigwa Lubuga, Urumwa and Ulyankulu and the rangelands with a diversity of wild edible fruits. The selection of study villages from the respective districts considered carefully the agro-ecological zones, upland/hill vegetation landscapes, lowland and riverine vegetation and wetland vegetation, and land uses, such as cultivated land, open grazing areas, and beekeeping zones. After being informed about the purpose of the study in all study locations, an official list of total households in each selected village was obtained from ward offices. Based on the total number of households in each study village, a 10% sample size of all households was preferred, and household questionnaire surveys were administered to obtain information about the diversity, use and degradation threats to wild edible fruits. Key informants such as forest officials from the Tanzania Forest Service (TFS), elderly members of the community and small processors of edible fruits were considered in the household sampled and interviewed to obtain in-depth ethnobotanical information about wild edible fruits.

### Data analysis

Ethnobotanical information collected from 244 heads of households was analysed using IBM SPSS v20 software and Ms Excel. Descriptive statistics was run to generate frequencies, percentages, and means of variables. In addition, to test the association between the socio-demographic information such as sex, age, household size, duration of stay, and education levels with the indigenous knowledge about wild edible fruits, the Pearson Correlation test (*r*) two–tailed test was computed. The ethnobotanical indices described below were determined to obtain information about utilisation frequency, use reports and cultural significance of the wild edible fruits.

#### Utilisation frequency (*f*)

Utilisation frequency (*f*) [41] was computed to quantify the use frequency of certain species listed by participants using the formula below:

1 $$f = \frac{Nm}{{Ni}}$$

where* f* represents the utilisation frequency, *Nm* is the number of informants mentioned in certain species, *Ni* represents the total number of informants. The higher the value of *f*, the more frequent the fruit species used.

#### Use Reports (UR)

Use Report (UR) information of a species mentioned by a participant within one use category was calculated by the formula below:

2 $${\text{UR}} = \sum \mathop \sum \limits_{i = i} {\text{UR}}ui$$

#### Cultural Importance Index (CI)

Cultural Importance Index (CI) expressed in [42], was computed in order to determine the diversity of use and the consensus of respondents using the equation below:

3 $${\text{CIs}} = \mathop \sum \limits_{{U = U_{1} }}^{{U_{NC} }} \mathop \sum \limits_{i = i1}^{iN} {\text{UR}}_{Ui/N}$$

where *N* is the total number of respondents, and NC is the total number of use categories. Thus, the Cultural Importance Index (CI) is the sum of the proportion of households that mention each of the use categories for a given species. The index indicates the spread of the use (number of respondents) of each species and the diversity of its uses. The relative importance of each fruit species is determined by additional use category. Therefore, the higher the CI value is an indicator of multiple uses.

#### Frequency of Citation (FC)

Frequency of Citation (FC) was computed by dividing the citation of a particular species mentioned by participants (C*i*) over the total number of times (*N*) that all species were mentioned, multiplying by 100.

4 $${\text{FC}} = \frac{Ci}{N} \times 100$$

## Results

### Socio-demographic and economic characteristics of the respondents

In this ethnobotanical study, a total of 244 local inhabitants from eight wards were interviewed to obtain information about the diversity of wild edible fruits, seasonal availability, their uses and threats. The respondents' socio-demographic and economic characteristics were considered in this study (see Table 2). The majority of respondents were female, and the minority were male. The average household size was 5.86 ± 3.34 (s.d.) persons. The majority of participants' households comprised 1–5 members, followed by 6–10, and the remaining households consisted of 11–15 members, 16–20 and a small number of households with more than twenty people. The age of the respondents ranged from 20 to 98, with the majority falling within the 20–29 age group. The results show that a more significant majority had completed primary education, compared to those who attained secondary level, higher education, and fewer people had never attended school. Married respondents made up the majority compared to respondents who were single, separated, widowed, and widower. Crop farming is a significant economic activity, followed by small or petty trades, livestock keeping and employment (Cf. Table 2).

**Table 2:** Sociodemographic characteristics of the participants in the study location

Sex	Frequency	Percent
Female	107	56.1
Male	137	43.9
*Education*		
Primary education	158	64.8
Secondary education	23	9.4
Higher education	1	0.4
None	62	25.4
*Marital status*		
Married	200	82.0
Single	19	7.8
Separated	2	0.8
Widow	11	4.5
Widower	12	4.9
*Households size*		
1–5	136	55.7
6–10	90	36.9
11–15	15	6.1
16–20	1	0.4
> 20	2	0.8
*Occupation*		
Crop farmers	195	72.8
Livestock keepers	18	6.7
Crop and livestock farmers	24	9
Small or petty business	26	9.7
Employed	5	1.9

### Knowledge about wild edible fruits, seasonal availability and diversity

The respondents reported 27 wild edible fruits and the ethnobotanical information recorded about these plants, including their vernacular names, seasonal availability, habitat, life form and use category. Of the respondents interviewed, 94.3% possessed strong knowledge of wild edible fruits found in their area, whereas 5.7% lacked knowledge. A significant association was found between socio-demographic information and the knowledge of wild edible fruits. Pearson product correlation of knowledge about wild fruits and household size was low and statistically significant (*r* = 0.221^**^, *p* < 0.001). The knowledge about wild fruits and sex was very low and negatively statistically significant (*r* = − 0.128^*^, *p* < 0.048). No correlation was found between the knowledge about wild fruits and the socio-demographic variables such as age, education levels, length of stay and marital status. There was a mean difference between the age of the respondents and knowledge of wild edible fruits (Table 3). Respondents aged 20–29 had better knowledge about wild edible fruits compared to other age cohorts.

**Table 3:** Mean age of the respondents

Age of the respondents	Mean	N	Std. error of mean
20–29	1.0755 ± 0.26668 (s.d.)	53	0.03663
30–39	1.0000 ± 0.00000 (s.d.)	47	0.00000
40–49	1.0750 ± 0.26675 (s.d.)	40	0.04218
50–59	1.0732 ± 0.26365 (s.d.)	41	0.04118
60–69	1.0667 ± 0.25371 (s.d.)	30	0.04632
70+	1.0606 ± 0.24231 (s.d.)	33	0.04218

Of the wild edible fruits reported, 64% were deciduous shrubs or small trees, 21% were shrub trees, and 15% were evergreen or deciduous trees. The results show that 56% of wild edible fruits were harvested from June to August after the rainy season, 33% between December and May during the rainy season, and 11% from September to November before the rainy season. Local people harvested various wild edible fruits at a considerable distance from homesteads; the locals had to trek to locations such as hilly areas, and bottomlands such as wetlands. Only a few species, including *Vitex mombassae* Vatke, *Tamarindus indica* L, and *Strychnos spinosa* Lam., were collected near homesteads since the local inhabitants are conserving and preserving them effectively.

### Use reports, utilisation frequency and cultural significance

**Fig. 2:**
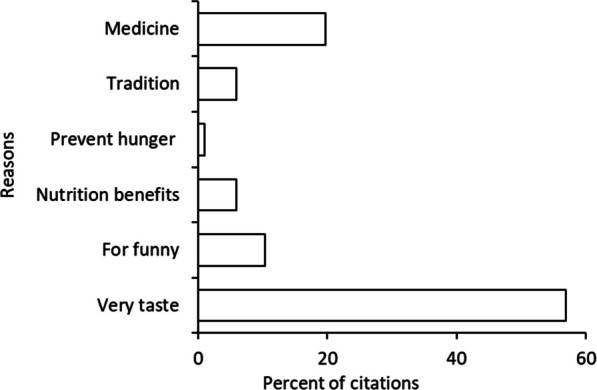
Reasons for utilising wild fruits

The survey results indicated that out of 1411 citations, the top most cited wild edible fruit with higher use reports was *Vitex mombassae,* with 276 citations, followed by *Strychnos spinosa*, with 228 citations, *Phyllogeiton discolor* (Klotzsch) Herzog., with 175 citations, *Vitex payos* (Lour.) Merr. with 150 citations, *Tamarindus indica* L. with 137 citations, *Friesodielsia obovata* (Benth.) Verdc*.,* with 136 citations, *Vangueria infausta* Burch., with 111 citations, *Parinari curatellifolia* (Planch. ex Benth.) with 61 citations, *Landolphia parvifolia* K.Schum*.,* with 54 citations and *Microcos conocarpa* Burret with 53 citations (Table 4). The higher cultural significance (CI) obtained in the findings denotes the multi-functionality of the fruits, mainly in the food and medicinal categories. Most participants in all study areas agreed that fruit species were considered culturally significant. In terms of utilisations, the most wild edible fruits with higher utilisation frequency (*f*) include *Vitex mombassae* (*f* = 0.84), *Strychnos spinosa* (*f* = 0.82), *Vitex payos* (*f* = 0.56), *Phyllogeiton discolor* (*f* = 0.45), *Vangueria infausta* (*f* = 0.45), *Tamarindus indica* (*f* = 0.38), *Parinari curatellifolia* (*f* = 0.25), *Landolphia parvifolia* (*f* = 0.22) and *Microcos conocarpa* (*f* = 0.22) (Table 4). The reasons given by the participants for taking the wild fruits included views that they were tasteful, had therapeutic properties, were traditionally conditioned, fun, nutrient-dense, and could reduce hunger (Fig. 2).

**Table 4:** List of wild edible fruits utilised by the locals in Tabora rural, Uyui and Sikonge districts and the different indices

Vernacular name	Scientific name	Botanical family	Life forms	Voucher number	Frequency food use	FC	*f*	Frequency medicine use	UR	*CI*
Mpunguswa	*Grewia flavescens* Juss	Malvaceae	Scrambling shrub	MEM 01	35	2.48	0.14	5	43	0.2
Msingilwa	*Flacourtia indica* (Burm.f.) Merr	Salicaceae	A deciduous shrub or small tree	MEM 02	8	0.57	0.03		8	0.0
Msungwi/Mtalali	*Vitex mombassae* Vatke	Verbenaceae	A small deciduous tree	MEM 03	205	14.53	0.84	56	276	1.1
Bonga pori/ Ungoungo	*Landolphia parvifolia* K.Schum	Apocynaceae	An evergreen climbing shrub	MEM 04	54	3.83	0.22		54	0.2
Mkwadunda	*Hexalobus monopetalus* (A.Rich.) Engl. & Diels	Annonaceae	A deciduous shrub or small with a scattered spreading canopy	MEM 05	40	2.83	0.16		40	0.2
Sekela	*Antidesma venosum* E.Mey.ex Tul	Phyllanthaceae	an evergreen to semi-deciduous tree or shrub	MEM 06	3	0.21	0.01		3	0.0
Nguwalu	*Vangueria infausta* Burch	Rubiaceae	A deciduous tree	MEM 07	111	7.87	0.45		111	0.5
Msaada/Saada	*Vangueria madagascariensis* J.F.Gmel	Rubiaceae	A shrub or small deciduous tree	MEM 08	7	0.50	0.03		7	0.0
Mgogwa	*Sclerocarya birrea* (A.Rich.) Hochst	Anacardiaceae	A deciduous tree	MEM 09	8	0.57	0.03		8	0.0
Msalasi/Salasi	*Friesodielsia obovata* (Benth.) Verdc	Annonaceae	Scrambling shrub or very small tree	MEM 10	106	7.51	0.43	22	136	0.6
Tundwa	*Ximenia americana* L	Olacaceae	A semi scandent bush-forming shrub or small tree	MEM 11	19	1.35	0.08		19	0.1
Makulwa/Mkome	*Strychnos pungens* Gagnep	Loganiaceae	A deciduous to sometimes evergreen tree	MEM 12	8	0.57	0.03		8	0.0
Mtonga	*Strychnos spinosa* Lam	Loganiaceae	A deciduous tree	MEM 13	199	14.10	0.82	14	228	0.9
Nkonze	*Manilkara mochisia* (Baker) Dubard		A shrub or small tree	MEM 14	33	2.34	0.14		33	0.1
Mkoma	*Grewia bicolor* Juss	Tiliaceae	A multi-stemmed shrub or small tree	MEM 15	5	0.35	0.02	1	6	0.0
Mwange/Mwage	*Strychnos spinosa* Lam	Loganiaceae		MEM 16	18	1.28	0.07		18	0.1
Ukwaju	*Tamarindus indica* L	Fabaceae		MEM 17	93	6.59	0.38	37	137	0.6
Gogondi	*Phyllogeiton discolor* (Klotzsch) Herzog	Rhamnaceae	Evergreen tree with drooping twigs	MEM 18	111	7.87	0.45	56	175	0.7
Mkamu	*Canthium burttii* Bullock	Rubiaceae	A shrub or a small tree	MEM 19	42	2.98	0.17		42	0.2
Mtowo	*Thespesia garckeana* F.Hoffm	Malvaceae	A small shrub or semi-deciduous tree	MEM 20	13	0.92	0.05	1	15	0.1
Mpulu	*Vitex payos* (Lour.) Merr	Verbenaceae	A shrub or small tree with a rounded crown	MEM 21	2	0.14	0.01		2	0.0
Mfulu genge	*Vitex payos* (Lour.) Merr	Verbenaceae		MEM 22	136	9.64	0.56	4	150	0.6
Mtinje	*Lannea humilis* (Oliv.) Engl	Anacardiaceae	A deciduous shrub	MEM 23	1	0.07	0.00		1	0.0
Muyongoyongo	*Multidentia crassa* (Hiern) Bridson & Verdc	Rubiaceae	A deciduous shrub or small tree	MEM 24	1	0.07	0.00		1	0.0
Mpundu	*Strychnos innocua* Delile	Loganiaceae		MEM 25	39	2.76	0.16		39	0.2
Mbula/Mbola	*Parinari curatellifolia* (Planch. ex Benth.)	Chrysobalanaceae	An evergreen shrub or tree with a dense, mushroom-shaped crown	FMM4209	61	4.32	0.25		61	0.3
Ndati	*Microcos conocarpa* Burret	Malvaceae subfamily Grewioideae	A deciduous shrub	FMM4212	53	3.76	0.22		53	0.2

*CF* Frequency of Citation, *f* utilisation frequency, *UR* Use Reports, *CI* Cultural Importance index

*CI* Cultural Importance index was computed based on participant consensus; CI index indicates the spread of the use (number of respondents) of each species and the diversity of its uses. The additional use category determines the relative importance of each fruit species; therefore, the higher the *CI* value is an indicator of multiple uses

**Fig. 3:**
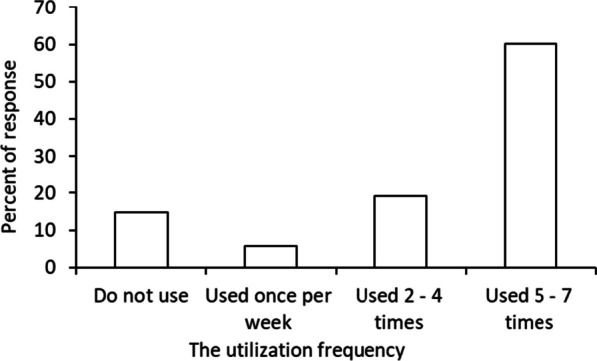
Utilisation of wild fruits weekly

The local inhabitants in the study areas consumed diverse wild fruit species daily as food. The use reports (UR) showed that almost all wild fruits recorded were commonly eaten fresh. In addition, *Tamarindus indica* and *Phyllogeiton discolor* species were cooked alongside traditional dishes such as oatmeal (porridge). The percentage of participants who cited using wild fruits more than five times a week was higher, followed by those who use them two to four times a week and once a week (Fig. 3). The findings show that the usage of wild fruits is related to the daily dietary needs and food supplements in the studied locations.

Species like *Strychnos spinosa* and *Tamarindus indica* were processed into local brew or juice. At the same time, *Phyllogeiton discolor* was dried and crushed to form nutrient-rich flour mainly used for children and ill persons. Most wild fruits have higher use frequency (Cf. Table 4) due to their multiple uses, such as food, medicine and cultural purposes.

Besides the food use category, the local participants in the study areas cited the species of wild fruits with pharmacological applications. A total of 10 medicinal fruit species used for therapeutic categories were documented (Table 5). The local participants in this research listed the different ailments treated using wild edible fruits and other ingredients. Among the ailments treated includes diabetes, anaemia, respiratory tract infections (RTI) and cough, pneumonia, urinary tract infections (UTI), constipation, Intestinal inflammation and syphilis, general stomach and constipation problems, gynaecological problems such as infertility, menstrual cycle problems and over bleeding issues (Table 5).

**Table 5:** Top most wild edible fruits with therapeutic uses in Tabora rural, Uyui and Sikonge districts

Species	Disease cured	UR	Percent	Literature supporting
*Phyllogeiton discolor*	Diabetes health problems, anaemia and gum problems	56	30	[57]
*Vitex mombassae*	Cough and respiratory tract infections	56	30	[46]
*Strychnos spinosa*	Stomach problems	14	8	[58]
*Friesodielsia obovata*	Gynaecological problems	11	6	Not reported
*Tamarindus indica*	Urinary Tract Infection, stomach and constipation	37	20	[46]
*Grewia flavescens*	Cough, pneumonia and bacterial throat infection	5	3	Not reported
*Vitex payos*	Diabetes health problems, headache	4	2	[25]
*Thespesia garckeana*	Chest pains, cough, infertility, liver problems and sexually transmitted infections	1	1	Not reported
*Grewia bicolor*	Anaemia, Intestinal inflammation and syphilis	1	1	[59]
*Parinari curatellifolia*	Stomach problems, reproductive disorders, anaemia and diabetes	2	1	[58]

*UR* Use Reports represents quotation frequency of species *i* mentioned by participant *N* and the consensus about its use importance

The dependence on natural medicine amongst the local participants is due to a lack of modern medical amenities and an inability to pay for modern treatment services where available. According to the survey results, out of 10 species cited (Cf. Table 5), *Phyllogeiton discolor*, *Vitex mombassae* and *Tamarindus indica* are the species with high use reports and are used most frequently due to their versatility in treating various illnesses. The medications of the cited species are mixed with tea, milk, or hot water; a patient can take medication three or four times a day until they feel better. A patient may be prescribed a dosage of 250 to 500 millilitres of a pharmaceutical mixture depending on one’s illness condition. Given the pharmacological function of the recorded wild fruit species in this ethnobotanical survey, the use of wild fruits for pharmacological purposes is essential because it influences conservation. Admittedly, these medicinal species are culturally valued in the studied locations.

### Threats to wild edible fruits

**Fig. 4:**
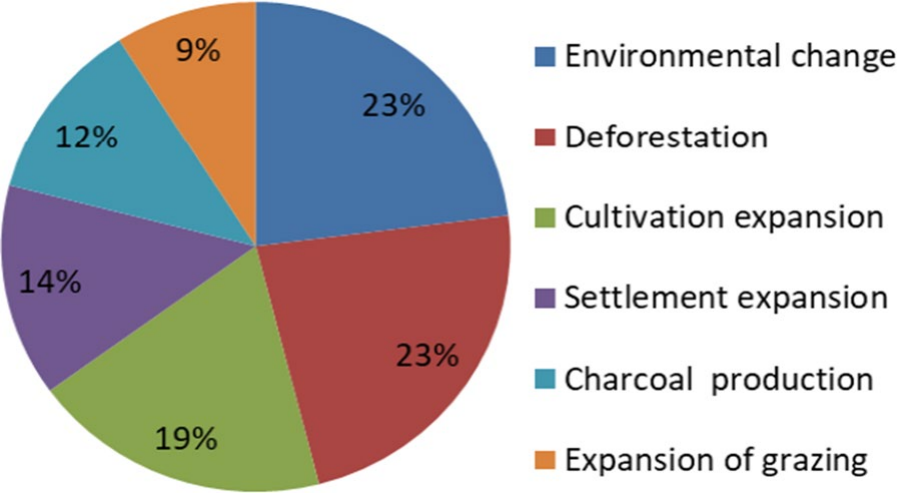
Threats to wild edible fruits in the study locations

According to respondents from all study villages, natural causes and land use practices threatened wild edible fruits in the study locations. The main threats to wild edible fruits and their abundance mentioned by the respondents were environmental change, deforestation and cultivation expansion with more citations, settlement expansion, charcoal production and expansion of grazing (Fig. 4). According to the respondents, the results showed that from 1990 until 2023, 85% of locals cited that there had been less of both variety and quantity of wild fruits, compared to 12% who cited no change and 3% cited that wild fruits were easily found. The majority 90% of participants cited that environmental change, such as climate has impacts on wild edible fruits, compared to 2% who disagreed and 8% were unsure.

The respondents cited fruit species that have diminished in abundance, including *Parinari curatellifolia*, with more citations, followed by *Friesodielsia obovata*, *Vitex payos*, *Strychnos spinosa*, *Vitex mombassae* and *Microcos conocarpa* were hardly found (Table 6). Respondents' consensus on the diminishing rate is higher for very quick responses, compared to quick and slow ones (Table 6). According to the local inhabitants’ knowledge, they cited that the consumption of wild fruit from 1990 through 2023 has declined. The survey showed that 59% agreed that the consumption had decreased, compared to 36% who said it had stayed the same, and 5% who thought it had increased. The percentage of respondents who cited a decrease in wild fruit consumption confirmed that, among other things, deterioration of rural systems of forest resource conservation and the erosion of customs around the use of natural fruits by younger generations are reasons to blame. The loss of plant diversity is another factor mentioned, as it contributed to a decrease in the consumption of wild fruits between 1990 and 2023.

**Table 6:** Wild edible fruits that are less common in Tabora rural, Uyui, and Sikonge districts

Species	Frequency	FC	Extent of diminishing		
			Very quickly (3)	Quickly (2)	Slow (1)
*Grewia flavescens*	12	4.55		2	
*Vitex mombassae*	25	9.47	3		
*Parinari curatellifolia*	40	15.15	3		
*Thespesia garckeana*	5	1.89			1
*Friesodielsia obovata*	39	14.77	3		
*Manilkara mochisia*	7	2.65		2	
*Strychnos pungens*	7	2.65		2	
*Microcos conocarpa*	24	9.09	3		
*Strychnos spinosa*	26	9.85	3		
*Vitex payos*	34	12.88	3		
*Strychnos spinosa*	7	2.65		2	
*Strychnos innocua*	13	4.92	3		
*Berchemia discolor*	4	1.52			1
*Tamarindus indica*	3	1.14			1

*FC* Frequency of Citation confirms list of fruit species that have decreased from a period from 1999 to 2023

## Discussion

### Socio-demographic characteristics of the respondents and the knowledge of wild edible fruit

In this study, different socio-demographic characteristics were compared with the knowledge about wild edible fruits (Cf. Table 2). Research shows that socio-demographic factors affect how people use and understand natural resources, making them a valuable source of indigenous knowledge for ethnobotanical studies like the ones conducted in the Chlef region of Algeria [43] and Bahia [44] in Salvador. The average number of members in a household obtained in this study is comparable with the Tanzania national 2022 population and housing census [45]. However, the findings could not reveal its influence on the wild fruits utilisation. Illiteracy levels in the current study were very high as the majority of the respondents had completed primary education, and it is comparable to [46], as they found in their ethnomedicinal survey that 67% of the participants in Nsenda ward in Urambo District, central western Tanzania had attained primary education level. Surprisingly, with regards to age and the knowledge related to wild fruits, most respondents fall within the 20–29 age group and possess a good knowledge concerning wild edible fruits (Cf. Table 3). The findings signify that customary of utilising wild edible fruits in the study locations is deeply entrenched in the local culture. The reasons for that is, the younger generations in the study locations are the ones who are intensely involved in charcoal production. Thus, they have a higher likelihood of exploring and knowing fruit diversity in their locations. On the contrary, individuals aged 60–70 + possessed relatively moderate knowledge about the diversity of edible fruits in their locations. The situation is due to their inability to walk far from their homestead to obtain the fruits.

Also, the respondents' sex constituted the female majority who headed their households different from males. In contrast [47] reported a similar custom in the North West Province where many households were female-headed. There was a significant association between respondents' sex and wild edible fruits knowledge. Admittedly, female respondents have better knowledge of wild fruits because they often interact closely with nature in their daily activities. Women in the study locations frequently collect plants for firewood and other wild fruits as domestic activities than males, who are herding cattle, charcoal production and beekeeping activity. This is comparable to [48] meta-analysis on ethnomedicinal knowledge that was related with respondent’s sex. Furthermore, a significant correlation between household size and wild edible fruit knowledge was evidenced in the study locations. Furthermore, It is assumed that the education level attained can be associated with wild fruit knowledge. However, the findings revealed no significant correlation between the education levels of the respondents and their knowledge of edible wild fruits. This is similar to [49] study conducted in Kenya whereas they discovered that formal education was not significantly correlated with ethnomedicinal knowledge. Furthermore, the age, length of stay, marital status, and occupation of the respondents were also not correlated with ethnobotanical knowledge in the present study.

### Utilisation frequency, use reports and cultural significance related to wild edible fruits

The availability of wild fruits influences traditional plant use in the research areas. According to the survey's findings, all the recorded 27 wild edible fruit species have multiple uses such as food, medicine and cultural practices. Wild fruits' diversity and seasonal availability are important components in this study because the local inhabitants have more selections to obtain their food and dietary needs. The diversity of natural habitats may be advantageous to different plant utilisation [49]. As revealed in the findings, most wild fruits were obtained from trees rather than shrubs. This finding aligns with [47] the ethnomedicinal survey conducted in the Urambo district of the Tabora region in central western Tanzania, where they reported that from wild fruits were obtained trees. The wild edible fruit species with higher utilisation frequency (*f*) and use reports (UR) were *Vitex mombassae*, *Strychnos spinosa*, *Vitex payos*, *Phyllogeiton discolor*, *Vangueria infausta*, *Tamarindus indica*, *Parinari curatellifolia*, *Landolphia parvifolia* and *Microcos conocarpa* (Cf. Table 4), the also considered cultural significant (CI) because of their multifunction uses. The reasons cited for consuming wild edible fruits in the study locations were: the fruits’ enjoyable taste, their medicinal applications, the locals’ tradition, nutrition benefits, and, less frequently, hunger prevention (Cf. Fig. 2). The local inhabitants agreed that all fruits recorded were consumed fresh because they are a good source of nutrients in their localities. The later observation echoes observations reported by [50] in eastern Bhutan where wild fruits were consumed fresh and regarded as part of their food. The nutritive value of wild fruits was reported by [30, 51, 52] in other parts of Tanzania and by [53] from Southern Africa.

Apart from being consumed fresh, *Tamarindus indica* serves as a special recipe that is cooked along with maize flour to make oatmeal (a local dish called porridge) in the study areas. *Berchemia discolor* is also pounded to create nutrient-dense flour cooked with maize flour to feed children and ill people. In urban areas, *Tamarindus indica* is largely processed to make juice or ice cream. In a lesser use, *Strychnos spinosa* is fermented to make local wine. The use of wild edible fruits in the study locations has a positive indication of the transmission of traditional knowledge for native plant conservation. The utilisations have resulted in the conservation of plant species such as *Vitex mombassae, Strychnos spinosa*, *Berchemia discolor*, *Vitex payos*, and *Tamarindus indica*. The seasonal obtainability of wild fruits has the potential to play a role in the food security of the local inhabitants year-round to meet their daily needs and nutrient supplements. Obtaining modern or exotic fruits is hard to come by, and when they are, the price is so high that the average person cannot afford to buy them. As a result, native edible fruits have higher ethnobotanical importance in the present study because they are a big source of vitamins. In the coastal forests of Tanzania, [51] reported the potential of wild fruits as dietary supplements to the local inhabitants.

In addition to having higher (*f*) and (UR) indices obtained in Table 4, the majority of wild fruit species recorded in this ethnobotanical study have low market values. This assertion is consistent with [54], who earlier observed that income from selling wild fruits is more for subsistence than as a capital asset for the household in the semi-arid Kondoa District of Tanzania. Very few wild fruits were sold in urban areas for cash generation. Yet, the local inhabitants are owing to a lack of markets. There have been similar constraints on the market for the native forest foods from the Eastern Arc Mountains areas of Tanzania [31]. In the research location, processing local fruits to add value and extend their shelf life is at an infant stage. This fact is unlike [53, 55], who claimed that products like jam, juice, and wine could be made from wild fruits. Short of understanding about value addition to process the fruits, lack of capital and markets are the setbacks hindering the processing of wild edible fruits in the study locations.

Besides the food use category, wild fruits are utilised as a natural medicine to cure various ailments. Tanzania's rural population mostly depends heavily on medicinal herbs as their primary health care [56]. The current study found that the absence of contemporary medical facilities and treatment services has taught the locals to rely on local fruits for medication. The top ten species used for treating ailments were recorded. *Berchemia discolor*, *Vitex mombassae* and *Tamarindus indica* have higher use reports because of their multifunction in treating diabetes, anaemia, cough, respiratory tract infections (RTI), pneumonia, urinary tract infections (UTI), constipation, intestinal inflammation and syphilis, stomach problems, gynaecological and infertility conditions (Table 5). The medicinal use value documented in the current study are in agreement with other ethnopharmacological studies, whereas [57] reported about cultural significance *Berchemia discolor* in Namibia and [46] documented the application of *Vitex mombassae* and *Tamarindus indica* for treating diarrheal in central western Urambo district of Tabora region in Tanzania. *Parinari curatellifolia* and *Strychnos spinosa* were other species reported by [58] in treating stomach, anemia and diabetes in Ujiji Kigoma of Tanzania. Similarly, [59] reported *Grewia bicolor* specie for treating hemorrhoids in the Urambo region of western Tanzania. Besides the fact that the cited wild fruits species are used by the locals in the study locations to treat different ailments, the study suggests the need for further investigation to understand their effectiveness. Based on the available literature, the active function is yet to be established. As documented in this study, pharmacological knowledge related to wild fruits utilisation is crucial. Therefore, it is important to promote experimental research relating to the safety and effectiveness of the therapeutic use in the study areas and in another places of Tanzania. Unfortunately, the knowledge and expertise in utilising wild fruits for therapeutic practices have been left to older generations, which poses the risk of losing this vital knowledge in the study locations. Similar observations were reported by [60] in eastern Tanzania and only a few people were familiar with the pharmacological use of wild plants in Morogoro.

### Threats to wild edible fruits and the indigenous knowledge

The diversity and abundance of wild edible fruits depend on the ecology of a given area and management. This study contends that various wild fruits are found in natural forests, which pastoral tribes or crop farmers primarily inhabit. Deforestation caused by increased need for grazing lands and farmland expansion severely threatens plant diversity in these places. Besides the cultural significance of the wild edible fruits cited, the study findings from all study locations found that numerous human activities have contributed to a loss of plants. In addition, field observations revealed that, settlement expansion and charcoal production have increased and putting wild fruit plants under threat of extinction (Cf. Fig. 3). In comparison with other studies [50] in eastern Bhutan, [61] in Ethiopia and [46] in Tanzania anthropogenic threats to wild fruits were reported as major threats to wild plants. Charcoal production in the study locations has caused severe loss of plant species with edible fruits. Illegal charcoal makers are clearing some valuable species (*Microcos conocarpa*, *Tamarindus indica*) to produce charcoal. According to the participants, the situation has been mainly amplified by the erosion of rural systems of landscape conservation amongst younger generations. Many younger informants regarded the consumption of wild fruits as an indicator of poverty and less importance. The similar conditions was reported by [62] in a study in northwest Yunnan province whereby many middle-aged generations ignored the consumption of native fruits. The younger generations in the study areas have a low interest in wild fruit use and even conservation. The situation has contributed to ignoring native fruits, as obtained in the current survey. Since the consumption of wild fruits has diverse benefits, such as nutrients and minerals, to food sources, the loss of their diversity impacts food security for local populations. As obtained in this study, fruit species such as *Parinari curatellifolia*, *Friesodielsia obovata*, *Vitex payos*, *Strychnos spinosa*, *Vitex mombassae* and *Microcos conocarpa* have disappeared considerably. The loss of diverse wild fruit species may contribute to the disappearance of traditional knowledge related to wild fruits. In addition to the effects of humans on wild fruits, environmental changes such as droughts and climate have considerably affected plant species' productivity. The local inhabitants explicated the loss condition and during the transect surveys it was revealed that *Vitex mombassae*, *Parinari curatellifolia, Microcos conocarpa* species have become less productive in the study locations. Based on the study findings, the researcher insists on sensitising locals about the worth of wild fruits in their areas and the potential for conserving the environment. Among the strategies suggested by informants to improve the conservation of wild fruit plants were making nurseries for domesticating wild species, prioritising the conservation of species with cultural importance, and providing public education on conserving wild fruit plants. There were also suggestions on promoting markets for native fruits and capacity-building regarding processing and storage.

### Implications of the study

This study has documented valuable information about the use, significance and threats to wild edible fruit in the miombo woodlands of Tanzania. The indices (*f,* CI, UR and CI) computed have created a better understanding of wild fruit species' diversity, use and cultural significance in the study areas. The present study found that the diversity of wild edible fruits has a crucial role in food and medicine. Wild edible fruits are a good source of nutrients and minerals for rural populations in the study areas because they do not have varieties in their daily diets. Admittedly, [63] showed that African baobab (*Adansonia digitata* L*.*) is a good source of nutrition and other health benefits. Of the total 27 fruit species recorded, ten species have medicinal importance. More important, in the present findings species such as *Friesodielsia obovata*, *Grewia flavescens* and *Thespesia garckeana* are reported for the first time and are useful against ailments (Cf. Table 5). The present study insisting the need to investigate their effectiveness against ailments and analysing the phytochemical and bioactivity potential to human health. Besides the cultural significance of the wild edible fruits recorded in the current study, there has been a downward trend in consumption from 1990 to 2023. The deterioration of rural systems of nature conservation amongst the younger generation has contributed to the decreasing culture of wild fruit consumption and related indigenous knowledge. Raising awareness about the nutritional benefits of wild fruits in the study locations is imperative in order to attract conservation interest. Investing in processing wild fruits into products such as jam, wine and juice, provision of capital, equipment and market soliciting are the options to value addition and can motivate the conservation of the wild fruits species. Policymakers should introduce formal or informal programs about indigenous fruits to inform younger generations about the importance of wild plants, sustainable use and management for future generations. Given agricultural technologies, there is potential for the domestication of some species of wild fruits that can be adapted to local environments, including those on the verge of going extinct. For the most part, understanding the phytochemical properties of the wild fruit species could provide better pathways to safe use [64].

### Limitations

This study has some limitations. First, the researcher could not record information about the availability index, frequency of use index, and parts of species used index. Recording this information could have been worthwhile in quantifying the cultural food significance index (CFSI), which considers various factors in evaluating a specific wild edible. Second, this study did not capture the quantity of harvested wild edible fruits, income generated from selling them, and their contributions to local economies. Besides the limitations the quality of this research has not been pretentious. The study has managed to document the indigenous knowledge related to the diversity of wild edible fruits, utilisation frequency, and cultural significance. Certainly, future research should quantify the CFSI index for better understanding of the multifunctionality of wild fruits.

## Conclusion and recommendations

This study has documented 27 species of wild edible fruits and the related indigenous knowledge in Tabora Rural, Uyui and Sikonge districts. It computed specific quantitative indices to determine wild edible fruits' use and cultural significance. It was found that the local inhabitants in the study locations rely on wild fruits for different purposes. More importantly, the study documented for the first time three *Friesodielsia obovata*, *Grewia flavescens* and *Thespesia garckeana,* beneficial species against ailments. Nevertheless, the study found that there has been a downward trend in utilisation of wild fruits from 1990 to 2023 and it has been caused by alteration of rural systems on nature conservation, expansion of human activities and environmental changes which have contributed to loos of some wild edible fruits near homesteads. It recommended sensitising the locals about the benefits of wild fruits and promoting consumption. The researcher supposes that markets soliciting and enhancing post-harvest storage and processing of fruits into other products is necessary to motivate the conservation of wild species and their habitats. The researcher also calls for further research on wild fruits' phytochemical and bioactive properties to ensure the safe consumption of these fruits. Lastly, the study insists on preserving indigenous knowledge associated with wild plants to ensure this valuable knowledge can continue for future generations.

## Data Availability

The data presented in tables and figures in this article will be made available on request.

## Abbreviations

°C: Celsius
Cf.: Confer
CFSI: Cultural Food Significance Index
CI: Cultural Importance Index
CONAS: College of Natural and Applied Sciences
*f*: Utilisation frequency
FC: Frequency of Citation
MUCE: Mkwawa University College of Education
NAFORMA: National Forest Resources Monitoring and Assessment of Tanzania Mainland
NTFPs: Non-Timber Forest Products
s.d: A standard deviation
TFS: Tanzania Forest Service Agency
UDSM: University of Da es Salaam
UR: Use Reports
WEPs: Wild Edible Plants
